# Fast Fatigue Life Prediction of Polymers Through Combined Constitutive Mathematical and AI-Based Modeling

**DOI:** 10.3390/polym18040456

**Published:** 2026-02-11

**Authors:** T. Barriere, S. Carbillet, X. Gabrion, C. Guyeux, S. Holopainen

**Affiliations:** 1 Marie and Louis Pasteur University, SUPMICROTECH, UTBM, CNRS, Institute FEMTO-ST, F-25000 Besancon, France; thierry.barriere@univ-fcomte.fr (T.B.);; 2 Marie and Louis Pasteur University, SUPMICROTECH, UTBM, CNRS, Institute FEMTO-ST, F-90000 Belfort, France; 3 Department of Civil Engineering, Tampere University, FI-33014 Tampere, Finland

**Keywords:** ultra-high-cycle fatigue, modeling, machine learning, minimal data, microstructural damage

## Abstract

The prediction of fatigue life is critical in the design process, and current models offer a viable alternative to costly and time-consuming experimental fatigue testing. The constitutive fatigue model used integrates low-cycle and high-cycle fatigue behavior. This model is grounded on the concept of fatigue damage evolution and incorporates a moving endurance surface within the stress space, eliminating the need for ambiguous cycle-counting methods. An interesting observation is that many polymers exhibit macroscopic fatigue characteristics, specifically, the form of the S−N curve similar to those observed in metals. Consequently, all fatigue model parameters were expressed in terms of the well-established Coffin–Manson–Basquin model parameters. However, the constitutive mathematical modeling itself is computationally time-consuming, particularly when applied to predict high-cycle fatigue across large design spaces. Therefore, the proposed model was utilized exclusively to generate high-quality data for training machine learning models that offer significantly improved computational efficiency. The high-cycle fatigue design of polymers and other ductile materials, traditionally dependent on expensive and time-consuming experimental methods, is now expedited through an advanced modeling framework that combines constitutive mathematical modeling with AI-based approaches.

## 1. Introduction

Due to their favorable properties (e.g., low cost, optical clearness, toughness, and low density), polymers represent highly important technological materials in automotive structural components (today, a third of the roughly 30,000 parts in cars are made of plastics) and aeronautic equipment (e.g., the cockpit canopy of the Lockheed Martin F-22 Raptor fighter), and polymers are also used as ingredients to improve the toughness of biocomposites and bulk metallic glasses [1,2,3]. Concurrently, fatigue failure of engineering components have been identified as a primary cause of significant financial losses and human injuries in traffic accidents [4,5,6]. Therefore, the development of high-performance materials and components intended for demanding fatigue applications is crucial. The development process can be significantly accelerated through model-based predictions, which have the potential to replace costly and time-consuming experimental fatigue testing. In other words, the model predictions enable rapid and systematic exploration of a wide range of material grades and loading scenarios.

Many existing fatigue models are based on fatigue-limit criteria, where the fatigue strength or limit is determined using a series of identical loading cycles. Alternatively, some models adopt cumulative damage theories combined with cycle-counting techniques [7,8,9]. However, defining a representative or standard cycle from complex loading spectra remains a significant challenge in practical applications. As an alternative approach, a continuum mechanics framework based on an incremental formalism of fatigue was introduced in the previous works [10,11,12,13]. In contrast to conventional cycle-counting methods, this framework defines damage evolution and the movement of the endurance surface in terms of stress increments rather than discrete stress cycles. This continuum mechanics framework is a consistent and unified formulation, allowing for the accumulation of damage and the application of stress-based fatigue limits under arbitrary loading histories. Particularly, the unified fatigue fracture model [14] proposed a (micro)structural–mechanical parameter to determine the crack formation depending on the asymmetry of the loading cycle.

However, constitutive mathematical modeling remains challenging and computationally time-consuming, particularly when applied to large scale-up design or the prediction of ultimately long fatigue lives. As a result, these sophisticated and deterministic models are increasingly being complemented or replaced by simpler and more efficient meta-models based on artificial intelligence (AI), which primarily require access to high-quality data [15,16,17]. Data-driven modeling approaches based on machine learning (ML, a prominent subset of AI) have demonstrated significant potential in fatigue analysis [16,18], as well as in simulating the nonlinear deformation behavior [19,20]. Laycock et al. [17], in this context, introduced the concept of Materials 4.0 referring to the current digital revolution in materials science that integrates ML with experimental methodologies. Correspondingly, in the fields of academic (1.0), theoretical (2.0), and computational (3.0) materials science, Wu et al. [21] proposed a ML framework to predict the macroscopic stress–strain behavior of ductile thermoplastic polymers. Their approach employs a stochastic Kriging machine model in combination with a genetic algorithm (GA) to train the machine model using only limited experimental data. Notably, Srinivasan et al. [22] demonstrated their ML framework for estimating the fatigue life of epoxy polymers and metal alloys using minimal experimental input. Xuan et al. [16] reviewed various hybrid physics-informed and data-driven ML (HPDML) methods for fatigue life prediction of metals: HPDML methods are based on a mutual interaction between a physically reasoned (constitutive) model and a ML method allowing for the design of the internal components of ML models faster and physically more transparently.

**Figure 1:**
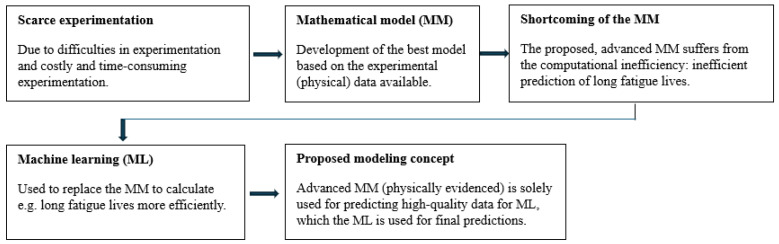
Flowchart for using the ML (for long-term fatigue), which is based on the experimental data and the high-quality data predicted by the advanced mathematical model.

However, a key limitation of previous studies is their reliance on costly experimental data, overlooking the optimal integration between experimentally acquired data, which are often incomplete due to resource constraints, and the model-predicted data, which can be generated in abundance. This work integrates the strengths of the traditional constitutive mathematical modeling with AI-based modeling into a unified framework, wherein the costly and often missing experimental data required for ML are substituted with high-fidelity model predictions derived from the available experimental data. The simulated or model-predicted data are of particular interest as a substitute for costly experimentally recorded data, whose value is expected to increase exponentially in the future [23,24]. This concept is illustrated in Figure 1. The difference to the previous studies is that the proposed concept benefits solely predicted model data to substitute missing experimental data. That is, no mutual interaction encoding between a physically reasoned (constitutive) model and a ML method is required. The proposed modeling framework, which combines constitutive mathematical modeling with AI-based methods, enables accurate predictions with reasonable computational times, even in the ultra-HCF range ($N>10^{8}$), which is especially critical for applications involving prolonged loading, such as tire durability [25] and fatigue life of various commercially significant materials [26,27,28].

This article continues by reviewing the fatigue testing methodology, the underlying fatigue model, and its calibration using experimentally validated Coffin–Manson–Basquin formulae analogous to those employed for metals. Finally, the article presents novel results and discusses the significance of incorporating mathematical model predictions into the ML framework.

## 2. Materials and Methods

The proposed approach for fast fatigue life prediction of ductile materials is general, but ductile plastics (polymers) were selected as a case study; the current polymer market size is immense with a valuation of approximately USD 660 billion for the most commonly used polymers. This figure is projected to grow to USD 1095 billion by 2032 [29]. Representative examples of polymer-based components subjected to fatigue loading can be found in the automotive and aeronautic industries [12,27,30]. Despite the critical role of polymer engineering, significant efforts are still needed to enhance their fatigue resistance [31,32] and to develop efficient and reliable simulation tools for predicting their fatigue behavior [9,12,33,34,35,36].

A PC polymer (Lexan^®^ 223R granulate, a density of 1.2 g/cm^3^) known for its high fatigue resistance was used in the fatigue experiments. The geometry of the flat, dog-bone-shaped tensile specimens, provided via injection-molding, conformed to ASTM standard [37] (type IV specimen). Optic 3D metrology (Alicona analyser, Bruker Alicona in Raaba/Graz in Austria) was employed to verify the high-quality surface and dimension accuracy of the specimens, ensuring homogenous stress and strain distributions and consequently, fatigue damage within the gauge section (web) of the specimens [11,12].

**Figure 2:**
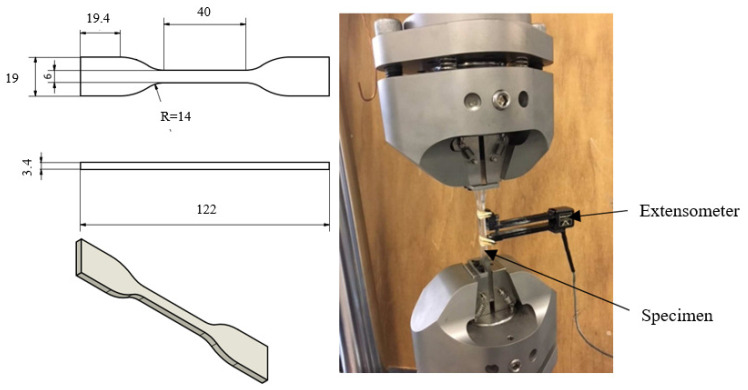
Geometry of the tensile test specimen (**left**) and the testing equipment (**right**).

Cyclic pulsating tensile tests were performed in accordance with standard [38], continuing until specimen rupture. An Instron Electropulse E10000 (Instron Division of ITW limited in Buchinghamshire, UK) with a load capacity of 10 kN and a maximum displacement of ±30 mm was used. An Instron 2620.601 extensometer with the capacity of 5 mm, i.e., 20% strain (glued onto the surface of the specimens’ web), was also used with data acquisition 1000 Hz, cf. Figure 2. The testing conditions were as follows:

- Force-controlled sinusoidal wave at f=5 Hz with stress ratios R=0.1 and 0.5;
- The maximum applied stress levels were 15, 37.5, 50, 75, 90, and 97% of the rupture (ultimate) stress, $\sigma_{u}=60$ MPa, for R=0.1 and 37.5, 50, 75, 90, and 97% for R=0.5.

Cyclic fully reversed tension–compression tests (R=−1) were also conducted to further refine the model parameters [12]. The fatigue process was also simulated at the frequency of 10 Hz [14], and the difference on strain to the frequency of 5 Hz was small. During testing, the axial force *F* and corresponding elongation *u* were recorded by the testing machine. Strain was defined as $\epsilon :=u/L_{g}$, where $L_{g}$ denotes the gauge length of the extensometer. The nominal (1st Piola–Kirchhoff) stress was calculated as σ=F/A, with *A* representing the original cross-sectional area of the gauge section. The deviation from the true Cauchy stress was minimal owing to the relatively small strain levels (less than 10%). However, the observed deformation behavior was clearly plastic, as evidenced by partially irreversible strains, a nonlinear σ−ϵ response, and the presence of ratcheting under asymmetric (R>0) force-controlled loading conditions.

## 3. Theory-Modeling

To investigate both low- and high-cycle fatigue (LCF and HCF) ranges, a capable simulation tool for fatigue must include an accomplished constitutive model which is able to predict (visco)elastic (HCF) and viscoplastic (LCF) deformation history including long-term creep and recovery, shape of the cyclic loading loops, and ratcheting. These material characteristics are particularly interesting for ductile plastics [11,12,34,39], and the models introduced in [11,36,40,41,42,43] include these elements.

The kinematics and constitutive formulation of the proposed model were detailed in [12]. The model is capable of capturing large deformations, as it is based on the multiplicative decomposition of the deformation gradient [36]. This framework enables the separation of local deformations into elastic and viscoelastic–plastic components, where the viscoelastic–plastic component represents the partially reversible micromechanisms. Macroscopically, this manifests as strain recovery upon unloading (viscoelasticity) and long-term phenomena such as creep strain and stress relaxation (viscoplasticity) [36]. The plastic response arises from irreversible, dissipative microstructural mechanisms, including specifically for PC polymer chain entanglement, slippage, and chain scission [44,45,46], which are often driven by the growth and coalescence of nano-/micro-scale voids [36,41,47,48].

### 3.1. Prediction of Fatigue Life

Considering the HCF range, the material exhibits primarily elastic macroscopic deformation, whereas in the LCF range, substantial plastic deformation develops. The transition from LCF to HCF typically occurs over a range of several thousand to tens of thousands of loading cycles. The fatigue model proposed in [12] encompasses both LCF and HCF ranges, and it is an extension of the HCF model originally developed for metals by [10]. Although metals exhibit dislocation micromechanisms, the model’s formulation is sufficiently general to be applicable to a broad class of ductile solids that exhibit similar macroscopic fatigue behavior; the model accounts for macroscopical asymptotical limits of fatigue life, namely, the endurance limit, and fatigue damage below this threshold is attenuated [10,11,33,41,49].

**Figure 3:**
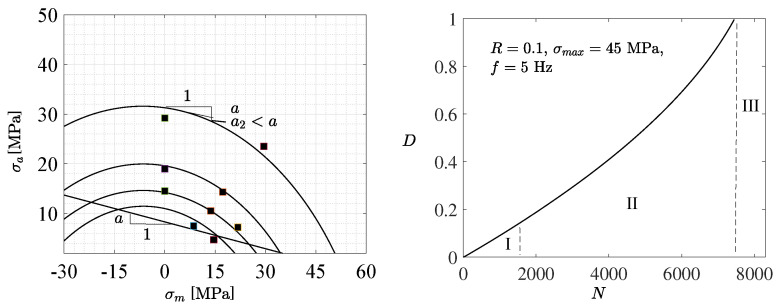
Predicted Haigh diagrams (besed on (1) for the PC) for 5000, 13,000, 25,000, and 40,000 cycles (**left**). The markers ■ denote the experimental data points. The linear approximation $∼\sigma_{a}\left(0\right)-aI_{1}$ ($I_{1}=\sigma_{m}$) representing the endurance limit ($\sigma_{a}\left(0\right)=\sigma_{0}∼8.3$ MPa) in the HCF range is shown, along with the interpretation of the model parameters a=0.18 and $a_{2}=0.014$ [12], which influence the curvature of the endurance surface. A systematic illustration of fatigue damage progression *D* is provided, showing stages I and II (as predicted by the model), and III (**right**).

To generalize the endurance limit, the proposed fatigue model introduces an endurance surface, denoted as β in stress space. When the stress state remains within this surface, no fatigue damage accumulates; conversely, damage initiation and progression occur when the stress state lies outside the endurance surface β [10,12]. As demonstrated in [12], the endurance surface of ductile polymers subjected to cyclic uniaxial loading (the stress oscillates between $\sigma_{m}-\sigma_{a}$ and $\sigma_{m}+\sigma_{a}$, with $\sigma_{a}$ and $\sigma_{m}$ denoting the stress amplitude and mean stress, respectively) is

(1) $$\begin{array}{c} {\left(\sigma_{a}+\frac{1}{2a_{2}}\right)}^{2}+{\left(\sigma_{m}+\frac{a}{2a_{2}}\right)}^{2}=K=\frac{1+a^{2}+4a_{2}\sigma_{0}}{4a_{2}^{2}} \end{array}$$

where $\sigma_{0}$ is the endurance limit and *a* and $a_{2}$ are material parameters with clear physical interpretations, cf. Figure 3(left). The proposed expression (1) defines a Haigh diagram specifically tailored for polymers and constitutes an advancement over the classical Gerber’s rule (1874), which, although historically significant for ductile materials (plastics), fails to accurately capture the asymmetry between tensile and compressive loading. The polymer-specific Haigh diagram derived from (1) is illustrated in Figure 3(left).

### 3.2. Fatigue Damage Evolution

**Figure 4:**
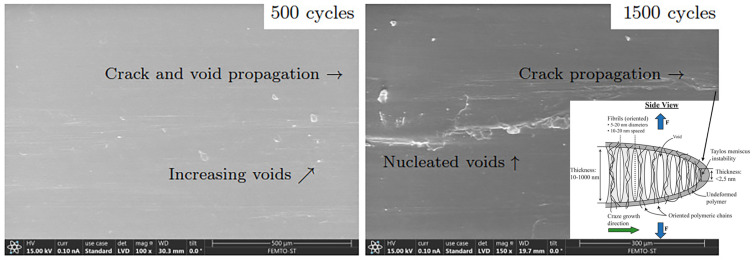
Initial microstructural evolution at 500 cycles (stage I, left) and 1500 cycles (stage II, right) under loading conditions of R=0.1 and $\sigma_{\max}=45$ MPa (75% of the ultimate tensile strength, $\sigma_{u}$). Scanning electron microscope (SEM) imaging (FEI Quanta 450 W EDS EDAX) was used. The inset (right) highlights the failure mechanism occurring at the crack tip.

Microstructural characteristics explain the long-term stable fatigue and deformation behavior of ductile materials before complete sudden failure (rupture). The stable phase governs typically more than 90% of the total fatigue life [11,33,41,49,50] and comprises the increase in nanoscopic voids [40,43,51]) and microcracks (primary stage I) to form macrocracks (secondary stage II) [47,52,53]; see Figure 3(right) and Figure 4. The impact of increasing voids was included in the constitutive model [12,36]. Furthermore, crazing, characterized by chain disentanglement and the formation of fibrils (extended chain crystals or bundles bridging voids), has been identified as a key mechanism underlying plastic deformation at crack tips during fatigue failure in stages I and II [44,45,46,54,55]; see Figure 4(right). During final failure (rupture), unstable macro-crack propagation accelerates rapidly, corresponding to the tertiary stage III. The complete sequence of fatigue failure stages I and III, along with the predicted fatigue damage characterizing stages I and II, is illustrated in Figure 3(right).

The evolution of fatigue damage characterizing the progressive fatigue failure process was modeled using an evolution law based on a scalar-valued damage variable. This scalar representation is appropriate, as it effectively captures the dominant fatigue damage stages I and II, which typically account for a majority of the total fatigue life (more than 90%) [36,49,50,56,57,58]. The multiplicative composition of the damage evolution was adopted, as introduced in [12]:

(2) $$dD=\frac{K}{1-BD}\exp\left(L\beta \right)\exp\left(-\tilde{L}\left(\beta +\frac{\tilde{L}}{\kappa }\left(\exp(-\frac{\kappa }{\tilde{L}}\beta )-1\right)\right)\right)d\beta ,$$

where the first and second exponential terms describe the evolution of HCF and LCF ranges, respectively. The parameters *K*, *B*, κ, *L*, and $\tilde{L}$ are all positive and possess distinct physical interpretations. Specifically, *K* and *B* govern the initiation of fatigue damage, refined by *L* and $\tilde{L}$ in the HCF and LCF regions, respectively. The parameter κ characterizes the duration of the transition between the LCF region and HCF ranges (the larger κ, the longer the transition); κ reflects on average, at the macroscopic level, the underlying microstructural evolution, particularly the accumulation of microcracks before rupture (tertiary stage III, where unstable macrocrack propagation accelerates rapidly) [11,12].

### 3.3. Model Calibration

**Figure 5:**
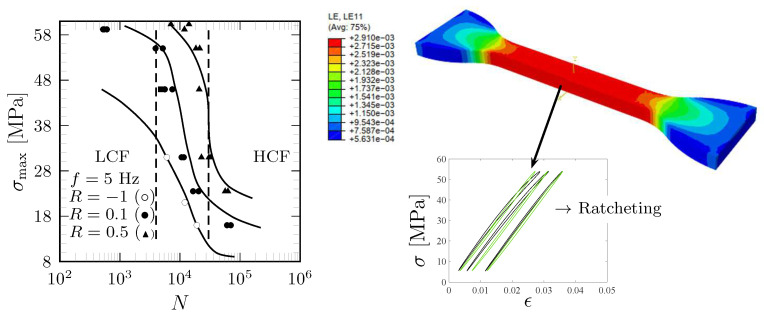
Predicted uniaxial S−N curves (fatigue lives, D=1) and experimental data (markers): R=−1 (the marker ∘), R=0.1 (•), and R=0.5 (▲) (**left**). FEM simulation (R=0.1, $\sigma_{\max}=0.9\sigma_{u}=54$ MPa, N∼80) demonstrating a homogeneous strain field (LE11=ϵ) in the gauge section of the specimen (**right**). The inset presents loops at the 40th, 400th, and 3100th cycles (prior to rupture) within the plastic LCF range (computed at a single material point). Black and green curves represent the experimental data and model predictions, respectively.

The model was implemented using the Intel^®^ Fortran application on the Puhti supercomputer (https://www.puhti.csc.fi/ (accessed on 20 December 2025). with Intel Xeon processors (Cascade Lake architecture), comprising two processors with a total of 40 cores operating at 2.1 GHz (excluding finite-element (FEM) simulations, to imitate a personal computer, only one CPU with the limited memory usage 4000 MB was used in the fatigue analyses). Based on the standardized specimen geometry [37], the strain distribution within the gauge section was assumed to be homogeneous. This assumption was verified through FEM simulations (UMAT in Abaqus), as illustrated in Figure 5(right). Consequently, fatigue simulations were conducted at a single representative material (integration) point within the gauge section, significantly reducing computational costs without compromising the fidelity of the results.

The constitutive model parameters used for predicting the macroscopic stress–strain behavior of ductile polymers (PC) were identified in [11,12]. The parameters related to the endurance function and fatigue damage evolution were calibrated using uniaxial cyclic test data, specifically from applied stress vs. number of cycles (S−N curves) shown in Figure 5(left). The observed similarity in macroscopic fatigue behavior (S−N curves) between polymers and metals motivated us to use the Basquin (HCF) and Coffin–Manson (LCF) empirical relations for polymers. Accordingly, the fatigue parameters (*K*, *L*, $\tilde{L}$, and κ) were formulated in terms of the univocal, well-established Basquin and Coffin–Manson parameters. These were complemented by the Ramberg–Osgood model parameters to capture the nonlinear plastic relationship between stress and strain, as detailed in [12].

## 4. Results

### 4.1. Predicted Results vs. Experimental Data

Both the measured and simulated S−N curves exhibit a characteristic pattern: an initially reduced negative slope at low cycle counts, followed by a relatively linear region with an increased negative slope, then transitioning to a diminished negative slope at higher cycle counts, and finally approaching a nearly horizontal asymptote in the ultimate HCF region, cf. Figure 5(left). Based on the curvature asymptotes of the S−N curves, it can be inferred that the LCF range encompasses fewer than approximately 4000...12,000 cycles, whereas the HCF range extends beyond approximately 35,000...60,000 cycles. The nonlinear nature of the S−N curves poses a significant challenge for modeling. Nevertheless, the proposed fatigue model, including the damage evolution (2), formulated as a composition of the LCF and HCF ranges, accurately captured the observed S−N behavior. In particular, the curvature parameter κ effectively characterizes the rate at which the transition between the asymptotes’ slopes of the LCF and HCF regions occurs. Furthermore, the viscoplastic constitutive model accounting for the effects of void growth demonstrated strong predictive capability in replicating the stress vs. strain loops and ratcheting strain behavior under asymmetric loading [12], as illustrated in Figure 5.

### 4.2. Predicted Data vs. ML

**Figure 6:**
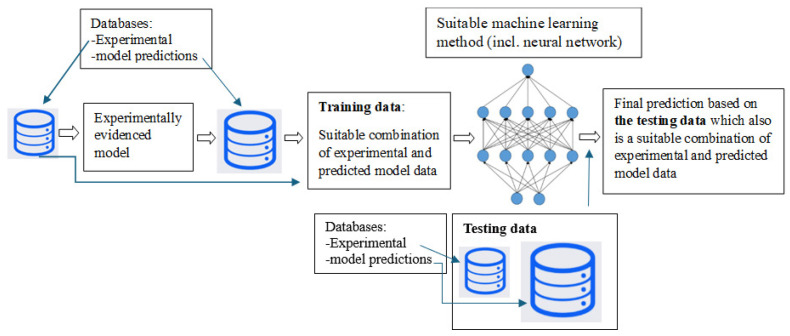
The proposed modeling concept based on ML boosted by high-quality data driven by advanced (explicit) design modeling. The Pearson correlation between two variables is also demonstrated (bottom left).

The following discussion addresses a common challenge in research: the limited availability of costly experimental data required to investigate a comprehensive range of conditions. In the present study, history-dependent data for long fatigue lives exceeding 40,000 cycles were unavailable. To address this, high-quality model data predictions generated by a calibrated, microstructurally informed constitutive model were employed to substitute the missing experimental data. This approach enables the construction of as-built models of real-life components [59] and facilitates the development of digital (numerical) twins, virtual counterparts of physical testing environments that dynamically mirror the behavior of their physical equivalents [60]. Figure 6 illustrates the proposed ML-based framework, in which the algorithm is trained and tested using a combined dataset comprising both the available experimental data and high-quality model-generated data. Accordingly, within this framework, the advanced mathematical model is employed exclusively for generating high-fidelity training data for the ML algorithms.

Maximum stress $\sigma_{\max}$ and fatigue damage *D* as a function of the number of cycles *N* were selected as the target variable to be predicted using ML. Due to the complex and highly nonlinear dependence of damage on the stress and strain histories, the choice of an appropriate ML approach is constrained. Traditional unsupervised methods, such as linear regression (LR) or least absolute shrinkage and selection operator (LASSO) [61], are inadequate for capturing such nonlinear behavior. Instead, more advanced supervised methods, particularly support vector regression (SVR), are preferred. SVR is a widely adopted extension of support vector machine (SVM) [62], particularly well-suited for modeling complex and weak nonlinear relationships among input variables [63,64] and minimizing prediction error through margin-based optimization techniques [65]. Supervised methods (including SVR), which can be implemented using neural networks, offer several advantages, including the absence of prior assumptions (based solely on data), high adaptability, and computational efficiency. However, supervised ML methods also present certain limitations [21]: [1] first, their predictive accuracy is highly dependent on the quality of the training data, which is often constrained by the high cost and limited availability of experimental measurements. [2] Whereas both experimental observations and numerical simulations commonly exhibit Gaussian distribution characteristics [66], many ML methods do not inherently model or replicate this statistical behavior effectively.

**Figure 7:**
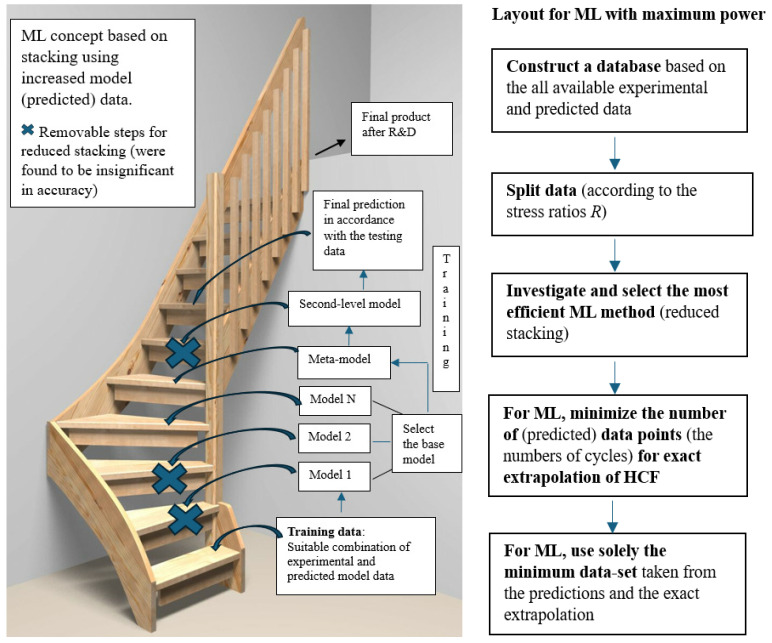
Schematic of the stacking method, which uses an increased amount of predicted data in relation to costly experimental data (**left**). Removed steps for the reduced stacking are also illustrated. Optimized procedure for ML to minimize the amount of data and calculation time (**right**).

To address the limitations of conventional ML methods, ensemble techniques have recently gained prominence for achieving higher predictive accuracy [61]. Ensemble methods aim to combine multiple ML models to enhance the overall performance and generalization capability. Notable examples include gradient boosting regression (GBR) and stacking [61]. Among these, stacking is often considered the most accurate approach, as it involves training several base models and using their individual predictions as input features for a final meta-model, and then using their individual predictions. In the proposed stacking framework, both the training data (used for training the base models and the first-level meta-model) and the testing data (used for validating the base models, first-level meta-model, and ultimately the second-level meta-model) are derived from a strategically balanced combination of an abundant set of high-fidelity model-generated data and the available experimental data, as illustrated in Figure 7(left).

To improve the ML process further, it is important to investigate the variables’ importance using, e.g., the random forest (RF) algorithm [61]. However, the application of such an autonomous method is unnecessary here because the mutual importance between the variables is considered known through the predictive constitutive modeling. The Pearson correlation is the best-known correlation measure for this purpose (demonstrated in Figure 6). The most important fatigue parameters are the endurance limit $\sigma_{0}$ and the slope parameters *a* and $a_{2}$ (demonstrated in Figure 3(left)), representing the material variables and ranging between 8.2 and 8.4 MPa, 0.17–0.19, and 0.013–0.017, respectively, to reach high accurate predictions. The Pearson correlation ρ between these variables is very low (∥ρ∥ < 0.01 for the random sample with the size 10,000), indicating that these variables are essentially independent of each other, when the model parameters can be considered constant and the model results are stable and reliable.

### 4.3. Fast HCF Predictions: Reduced Stacking

**Figure 8:**
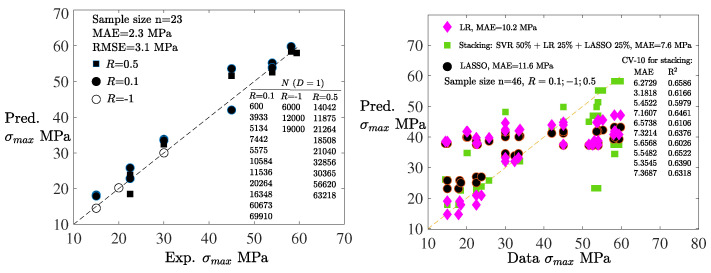
Comparison of the predicted (by the constitutive model) maximum stress values to the experimental values (the maximum stress decreases with increasing *N*) for the fatigue life (D=1) using a reference line (left). Comparison of the ML predictions to the data values: 50–50% combination (blending) of the constitutive model predictions and the experimental values shown on the right. Scattering of the results is due to the arrangement of data in accordance with the stress ratios: R=0.1;−1;0.5.

**Figure 9:**
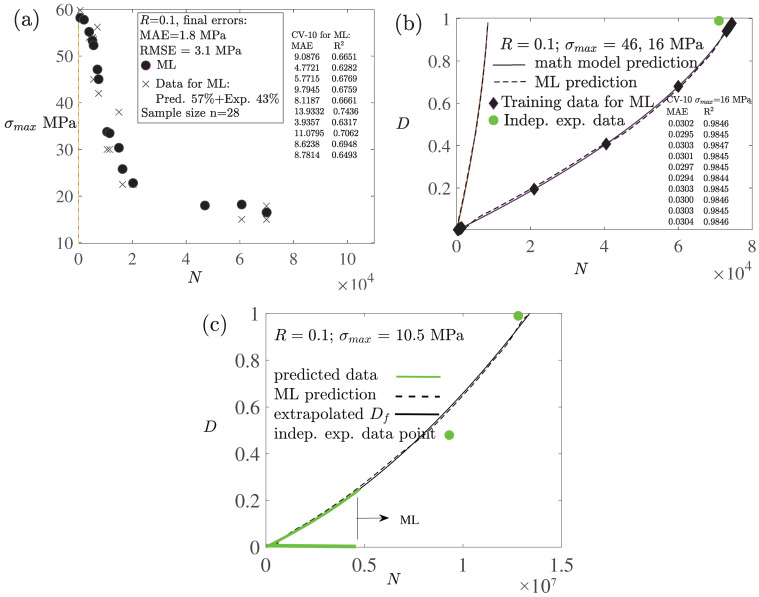
Prediction of the S−N curve (D=1) using the mathematical model and the ML via stacking based on the SVR (**a**). Predictions of fatigue damage *D* using the mathematical model (solid) and ML (dashed) (**b**). The total number of cycles *N* to failure, D=1, was validated experimentally (green markers; $\sigma_{\max}=16$ MPa as an example) and also the data points for training of the ML are shown (black markers ⧫). A quadratic fitted damage function $D_{f}$ and the ML prediction of HCF based on the $D_{f}$, and the predicted data (highlighted by the green color) which represents ∼30% of the expected fatigue life is also shown (**c**).

To further enhance particularly the prediction of HCF damage, a reduced stacking strategy was adopted, wherein the first-level meta-model was constructed using a single base model (SVR) employing random data splitting [64,67]. Notably, the inclusion of multiple base models offered minimal improvement in predictive performance. In the ablation study, three base models of LR, LASSO, and SVR were applied, and the two first were removed from the system: Figure 8(left) first shows the capability of the constitutive model to produce accurate data, and then Figure 8(right) evidences the increased accuracy of the SVR in relation to LR and LASSO. A notable error of the methods, which is due to the arrangement of data according to the stress ratios (R=0.1;−1;0.5), reveals the mutual capability of the methods. Moreover, the SVR as the first-level meta-model served also as the final predictive model because of its high accuracy, as shown in Figure 9a. This streamlined approach demonstrated in Figure 7(left) proved both computationally efficient and sufficiently accurate for assessing long-term fatigue damage and life. Figure 8(right) and Figure 9a show the 10-fold cross-validation used to estimate the ML model´s ability to generalize unseen data based on the SVR: Gaussian kernel, C (regularization), gamma (curvature), and epsilon were the hyperparameters with the values 1000, 10, and 0.01, respectively, (tuning iteratively by searching for 10 times magnitudes).

The prediction of fatigue damage *D* in addition to the S−N curve is crucial. In the present study on fatigue damage, where damage is a model variable, no direct (explicit) experimental data on damage evolution was available. However, the stress vs. strain relationship (σ−ϵ curve) affecting the full damage D=1 was experimentally validated, as shown in Figure 5 and Figure 8. Considering the number of predicted data points P=N/(f·Δt) (increments using the time increment Δt and frequency *f* generated by the full mathematical model). For instance, predicted data for N= 75,000 cycles (Δt=0.004 s, f=5 Hz) comprises almost four millions data points and time increments. Figure 9b shows the ML predictions when based solely on 0.3% of data points *P* (concentrated on the first and last 300 cycles). To identify this minimum dataset vs. maximum accuracy of the ML results, the number of data points was reduced by one tenth at a time. Despite the low data volume detailed in Table 1, the ML predictions show only a small error due to the smoothness of the predicted damage evolution. A scalability/performance analysis for the D−N relationship is summarized in Table 2. A conclusion is that majority of resources (CPU elapsed time) is needed for training, and the calculation time increases more than linearly when fatigue life increases, despite the reduced set of training data.

However, the primary challenge in predicting extreme HCF ($N>10^{7}$) lies in the significant cost and time associated with both experimental testing and also the mathematical modeling. As a result, comprehensive HCF data are typically unavailable. In contrast, ML methods offer significant computational efficiency. To address this limitation, the objective was to predict fatigue damage over a limited portion of the fatigue life, and to extrapolate the remaining damage response based on this partial dataset. Polynomial and exponential functions are suitable candidates for extrapolating fatigue damage response. In this approach, it is essential that the error between the function and the available predicted data remains minimal. It was observed that a quadratic polynomial function, $D_{f}=aN^{2}+bN$ (*N* is the number of cycles, and $a=2.6\times 10^{-15}$ and $b=4.0\times 10^{-8}$ are parameters), is accurate. The deviation between this polynomial function and the damage predictions from the constitutive model was found to be less than 0.5%. The proposed efficient methodology, which significantly reduces both the required data volume and computational time of HCF prediction, is illustrated in Figure 7(right). The proposed approach is particularly advantageous for very-HCF and ultra-HCF predictions, where conventional mathematical models are computationally intensive and time-consuming (in addition to experimental resources) [11,25,27,28]. In contrast, ML methods offer significant computational efficiency. However, the damage evolution increasingly relies on a fitted damage function which, given that direct experimental damage evolution data are not available, limits independent validation and predictive or extrapolative confidence in the ultra-HCF regime.

Figure 9c illustrates the predicted and experimentally validated data (highlighted in green), the proposed polynomial damage function $D_{f}$, and the corresponding ML prediction based on both the predicted data and $D_{f}$. The ML prediction aligns closely with the expected fatigue damage and fatigue life, even with the independent experimental data point for full damage, D=1 (note that the number of cycles *N* for D=1 corresponds to the experimentally observed specimen’s rupture). In the ML prediction, approximately 0.3% of the data points (concentrated on the first and last 300 cycles within 0–∼30% of the fatigue life) was required to define the D−N curve accurately. Additionally, a few supplementary points were extracted directly from $D_{f}$. Consequently, the total number of data points used amounted to only ∼0.3% compared to those that would be required by the mathematical model to predict the full fatigue damage and lifetime. The total CPU-elapsed time for the mathematical model, employing a maximum constant time increment of approximately 0.004 s within the implicit backward Euler integration scheme, was 67,800 s. In contrast, the average computation time for the ML prediction, with both training and testing phases, was ∼440 s (see Table 2), corresponding to only 0.65% of the computation time required by the full mathematical model. This study demonstrates that the proposed ML-based approach enables a huge decrease in both the number of data points (time increments) and the overall computation time.

## 5. Conclusions

**Figure 10:**
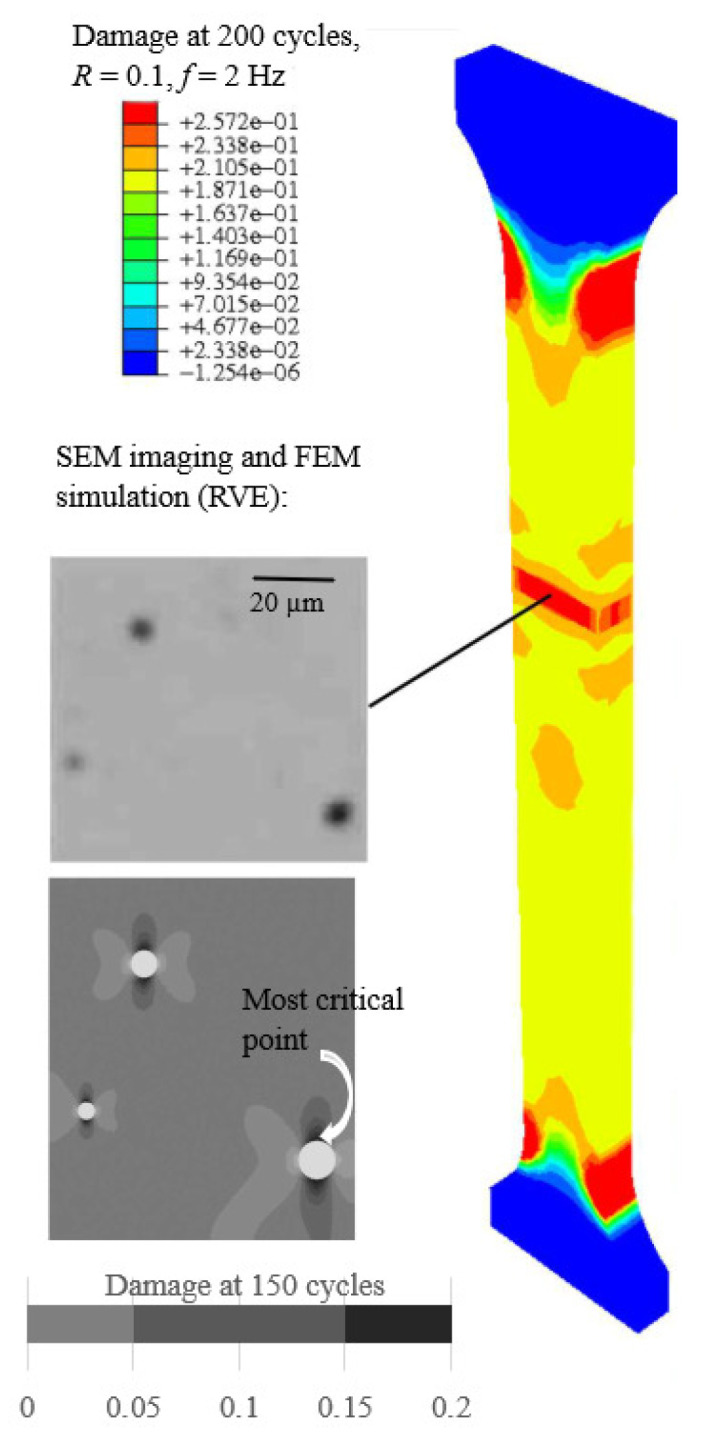
Localized fatigue damage ($\sigma_{\max}=97$% of the $\sigma_{u}$) caused by voids (in PC), based on the [12] model implemented in Abaqus/Implicit (**right**). Experimentally observed (SEM) region of voids and simulated damage in the corresponding RVE (**left**).

Due to the compact formulation of the proposed mathematical fatigue model and its systematic calibration procedure grounded in the Basquin (for HCF) and Coffin–Manson (for LCF) formulations [12], product development can be significantly accelerated through predictive modeling, enabling the creation of digital (numerical) twins and reducing the reliance on time-consuming trial-and-error testing procedures. The proposed approach is straightforward to implement and integrate as a built-in feature within finite-element method (FEM) packages. In this treatment, engineers must identify and select only the most critical material points (e.g., every ten thousandth point), while still performing calculations on several thousands points [68], as illustrated in Figure 10. In this context, the proposed modeling framework, which couples constitutive mathematical modeling with AI-based techniques, enables [1] accurate fatigue predictions within reasonable calculation times, even for ultra-HCF ($N>10^{8}$); [2] notably, the calculation (CPU-elapsed) time required for fatigue prediction at a single material point was reduced to just less 1% of that required by the full mathematical model; [3] the effectiveness of the proposed ML approach in the full fatigue analysis is remarkable:

According to Amdahl’s Law, the overall speedup of the calculation time can be increased to the order of 1/((1−F)+F/S), where *F* is the fraction of total CPU time spent on integration point calculations and *S* is the speedup factor achieved at a single integration point (>100 in this case). Using the moderate value F=0.9, the overall speedup of the calculation time is ∼10. The robustness of the approach could be further improved using deep ML, physics-informed neural networks (PINNs [69]), and use of parallel computing to reduce simulation times [70,71]. These advancements represent a significant step forward in improving the computational efficiency of HCF in future, while anticipating future breakthroughs in hardware, such as quantum computing [72].

## Figures and Tables

**Table 1:** Usage (blending) of the experimental and predicted data for the ML. In Figure 8(right) and Figure 9a, the portions represent solely the amount of data available (data points were randomly selected). In Figure 9b,c, the same portions were used for training and testing.

	Pred. Data %	Exp. Data, Explicit %	Exp. Data, Implicit % ^‡^
Figure 8 (right) .........	50	50	-
Figure 9a .........	57	43	-
Figure 9b .........	90	0 ^†^	10
Figure 9c.........	90	0 ^†^	10

^†^ Only two points: (D=0,N=0) and (D=1,N). ^‡^ σ−ϵ data during cycles used for the validation of the constitutive model.

**Table 2:** Scalability/performance analysis for the two D−N relationships shown in Figure 9b,c. The total sample sizes (predicted) are $P_{1}$ = 3,750,000 and $P_{2}=6.5\times 10^{8}$, respectively. The CPU elapsed time and the memory usage represent the average values.

	*N*	Sample Size	CPU Elapsed Time (s)	Memory Usage (MB)
Training .........	75,000	0.3% of $P_{1}$	1.9	3400
Testing .........	75,000	0.3% of $P_{1}$	0.01	3500
CV-10 .........	75,000	0.3% of $P_{1}$	0.02	3600
Training .........	13,000,000	0.3% of $P_{2}$	433	4100
Testing .........	13,000,000	0.9% of $P_{2}$	3.5	3600
CV-10 .........	13,000,000	0.9% of $P_{2}$	8.0	3700

## Data Availability

The source data supporting the findings of this study are available by the request. Specifically, the data underlying Figure 5(left) and Figure 8 are provided. The source data cover experimental and experimentally evidenced (predicted) model data.

## Nomenclature

ML	machine learning
N	numbers of cycles
f	frequency
R	stress ratio
$\sigma ,\sigma_{u}$	uniaxial (true) stress and its ultimate value
ϵ	uniaxial strain
HCF, LCF	High-cycle fatigue, low-cycle fatigue
$\sigma_{a}$	stress amplitude
$\sigma_{m}$	mean stress
$I_{1}\;$	first stress invariant
$a,\;a_{2}\;$	slope parameters of the Haigh diagram for low and high values of $\sigma_{m}$
*D*	fatigue damage
$\sigma_{0}\;$	fatigue strength or endurance limit
SEM	Scanning electron microscope
β	endurance function
K,L	fatigue damage parameters for HCF
$\tilde{L},\;\kappa \;$	fatigue damage parameters for LCF
$\epsilon_{r}\;$	ratcheting strain
SVR	Support vector regression
*P*	number of predicted data points
Δt	time increment
$D_{f}$	polynomial fatigue damage function
